# Non-destructive, continuous monitoring of biochemical, mechanical, and structural maturation in engineered tissue

**DOI:** 10.1038/s41598-022-18702-x

**Published:** 2022-09-28

**Authors:** Anne K. Haudenschild, Benjamin E. Sherlock, Xiangnan Zhou, Clay S. Sheaff, Jerry C. Hu, J. Kent Leach, Laura Marcu, Kyriacos A. Athanasiou

**Affiliations:** 1 Department of Biomedical Engineering, University of California, Davis, CA 95616 USA; 2 Department of Biomedical Engineering, University of California, Irvine, CA 92697 USA; 3 Department of Orthopaedic Surgery, UC Davis Health, Sacramento, CA 95817 USA

**Keywords:** Optics and photonics, Translational research, Tissue engineering

## Abstract

Regulatory guidelines for tissue engineered products require stringent characterization during production and necessitate the development of novel, non-destructive methods to quantify key functional parameters for clinical translation. Traditional assessments of engineered tissues are destructive, expensive, and time consuming. Here, we introduce a non-destructive, inexpensive, and rapid sampling and analysis system that can continuously monitor the mechanical, biochemical, and structural properties of a single sample over extended periods of time. The label-free system combines the imaging modalities of fluorescent lifetime imaging and ultrasound backscatter microscopy through a fiber-based interface for sterile monitoring of tissue quality. We tested the multimodal system using tissue engineered articular cartilage as an experimental model. We identified strong correlations between optical and destructive testing. Combining FLIm and UBM results, we created a novel statistical model of tissue homogeneity that can be applied to tissue engineered constructs prior to implantation. Continuous monitoring of engineered tissues with this non-destructive system has the potential for in-process monitoring of tissue engineered products, reducing costs and improving quality controls in research, manufacturing, and clinical applications.

## Introduction

Tissue engineering and regenerative medicine hold great promise for functional restoration of diseased and damaged tissues. Translating these tissue engineered solutions into clinical practice and commercialization faces several challenges including the inherent patient and cellular variability that affect the maturation rate of tissue formation^1^. In an effort to reduce this variability, the U.S. Food and Drug Administration (FDA) requires thorough characterization and quality control (QC) testing during both the development and manufacturing of all biological and tissue engineered products prior to clinical use^2^. Current QC methods that ensure the desired tissue biochemical and mechanical properties are achieved prior to implantation rely heavily on expensive, and time-consuming destructive testing^3^. Constructs evaluated by these methods are no longer suitable for implantation. It has been calculated that approximately 70% of the cost of tissue engineered goods can be attributed to QC efforts to satisfy FDA regulations^4^. QC in both scale-up and manufacturing processes is considered a major bottleneck in clinical translation and the President’s Council of Advisors on Science and Technology identified advanced manufacturing, including biomanufacturing, as an important aspect of keeping America competitive^2^. Continuous monitoring enables quick adjustments to manufacturing protocols, removal of under-performing samples, and predictive capabilities of product quality. Replacing destructive methods with non-destructive alternatives would reduce costs in research, meet FDA and international regulatory requirements for manufacturing safe, effective, and reproducible products, and provide novel and robust data through repeated measures of samples over time.

A range of non-destructive techniques have previously been employed to monitor changes in either the structure or biochemistry of tissue engineered constructs including Fourier transform infrared (FT-IR) spectroscopy, Raman spectroscopy, and magnetic resonance imaging but have limitations that preclude high-resolution, sterile, longitudinal monitoring of samples^5,6^. One promising approach for non-destructively characterizing the salient characteristics of tissue engineered samples is a multimodal system combining Fluorescence Lifetime Imaging (FLIm) and Ultrasound Backscatter Microscopy (UBM). FLIm is a fiber-based, label-free approach that uses endogenous tissue autofluorescence lifetime (LT) to probe biochemical features and has had previous success in the identification of cancerous tumors, cardiovascular disease, and articular cartilage degradation^7–9^. UBM provides high-resolution, full-depth structural information. Together, these imaging modalities can fully characterize the properties necessary for successful functional tissue engineering.

The focus of this work was to develop a system for non-destructive, in-process, and rapid assessment of tissue engineered products, using the manufacturing of tissue engineered cartilage implants as a model system.

To illustrate the ability of the multimodal system to detect differences in tissue biological, mechanical, and structural properties, we performed a growth factor study. The active form of transforming growth factor-$\beta$1 (TGF) was used to increase tissue maturation rate and mechanical properties. A novel latent form of TGF (LAP)^10^ that increases tissue engineered construct homogeneity^11^ was used to create unseen, internal changes in developing tissue structure.

**Figure 1:**
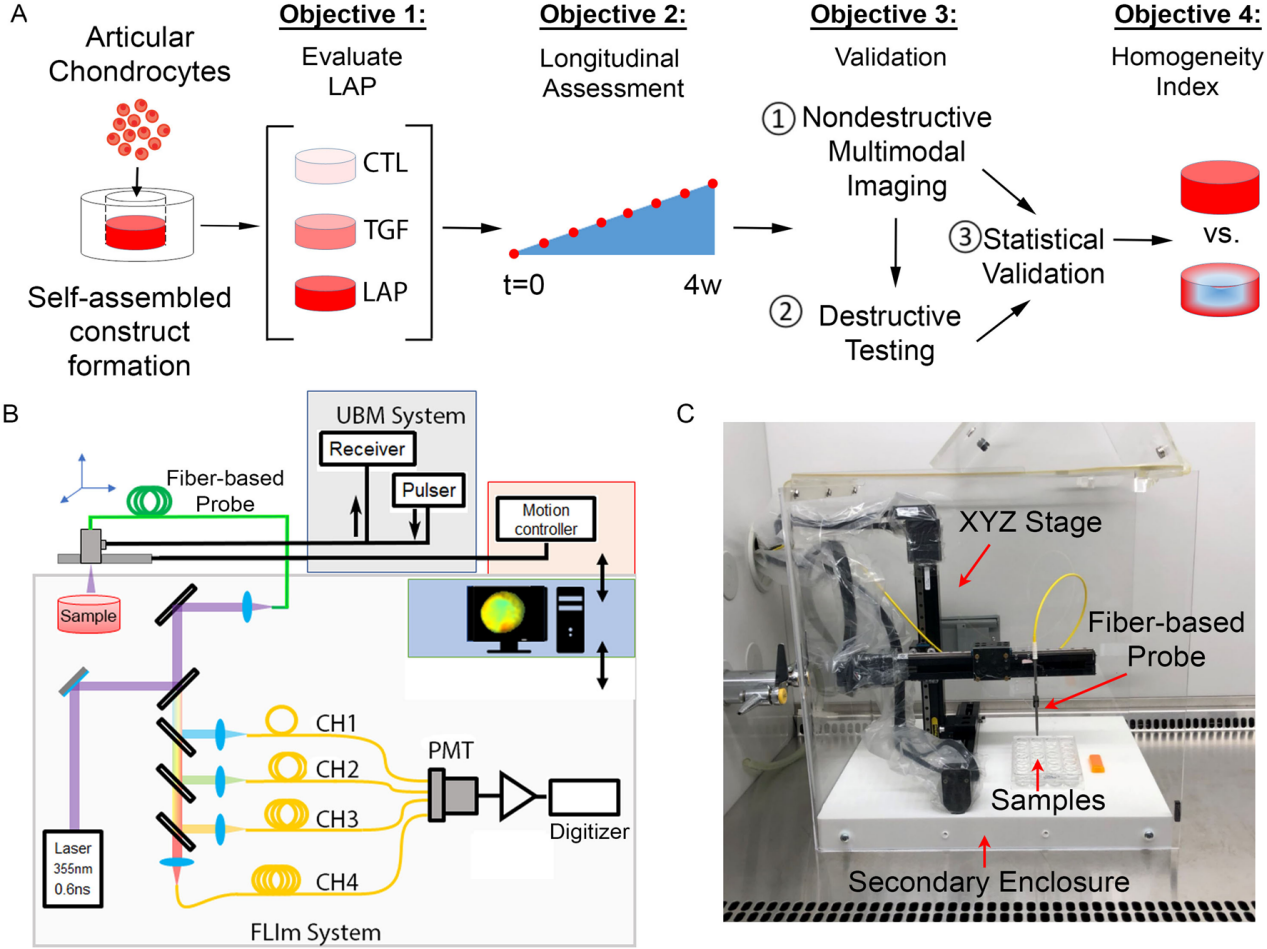
Experimental design and instrumentation. (**A**) Schematic overview of the study. (**B**) Schematic diagram of the multimodal imaging system. (**C**) Optical system interface in a standard biosafety cabinet allowed sterile monitoring of single tissue engineered constructs over time.

The specific objectives of this study were (1) to make a multimodal system optimized for assessing tissue engineered cartilage and perform longitudinal tracking of developing tissue maturation and homogeneity (2) to evaluate the system’s ability to detect improved tissue properties in a growth factor study, (3) validate optical parameters with gold-standard destructive assays, and (4) develop a novel imaging-based homogeneity index (Fig. 1a). We hypothesized that a multimodal system could quantify growth factor-induced improvements in tissue maturation and homogeneity and that an imaging-based homogeneity index combining biochemical and structural data could provide a novel, non-destructive assessment of tissue quality not currently available with destructive testing methods.

## Results

### Longitudinal assessment of constructs with FLIm detected increased tissue maturation

**Figure 2:**
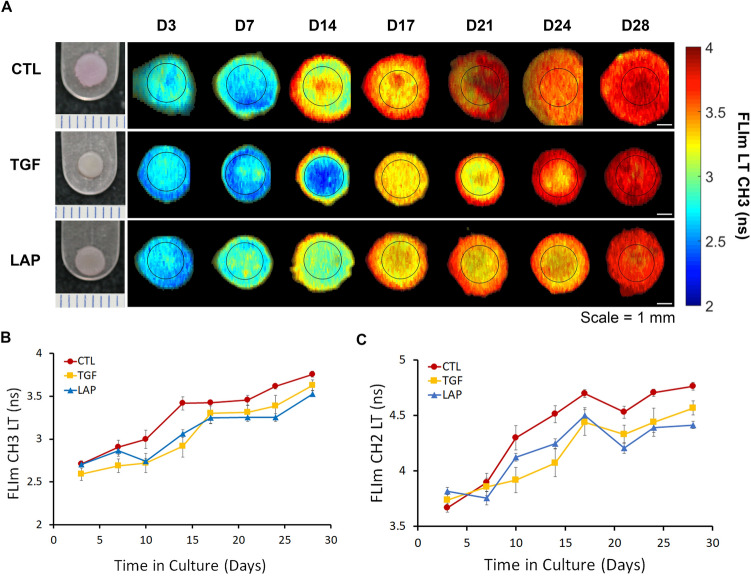
Longitudinal monitoring of constructs with FLIm detected LAP-induced tissue maturation. (**A**) FLIm assessment under sterile conditions for 28 days detected biochemical maturation in all 3 groups over time. (**B**) Quantification of FLIm CH3 LT and (**C**) FLIm CH2 LT showed increased LT with treatment and time.

The FLIm system was optimized for quantitatively monitoring tissue engineered constructs properties by combining label-free, sterile, and non-contact imaging with real-time data acquisition speeds. All data was normally distributed based on the Shapiro-Wilk goodness of fit test (*p* < 0.001). Non-destructive FLIm assessment, conducted under sterile conditions over 28 days, detected biochemical maturation in all three groups over time. Representative FLIm CH3 (532–565 nm) LT images from days 3 (D3) to 28 (D28) showed increased LT values over culture duration (Fig. 2a). Qualitative differences in the uniformity of the FLIm signal across the sample were observed. On Day 14, we observed large variations in FLIm CH3 LT values in CTL and TGF constructs while the LAP treatment produced more similar LT values across the sample (Fig. 2a). Both treatment and culture duration were found to significantly increase FLIm CH3 LT (*p* < 0.0001) (Fig. 2b) and FLIm CH2 (450–485 nm) LT (*p* < 0.0001) (Fig. 2c). Multivariable modeling showed that repeated FLIm measurements of longitudinal samples (imaged n=8 times) did not have a significant effect on mechanical properties (*p* = 0.64), biochemical content (*p* = 0.21), or FLIm LT values (*p* = 0.11) as compared to samples imaged only twice prior to destructive testing.

### Quantitative UBM detected a reduction in void formation

**Figure 3:**
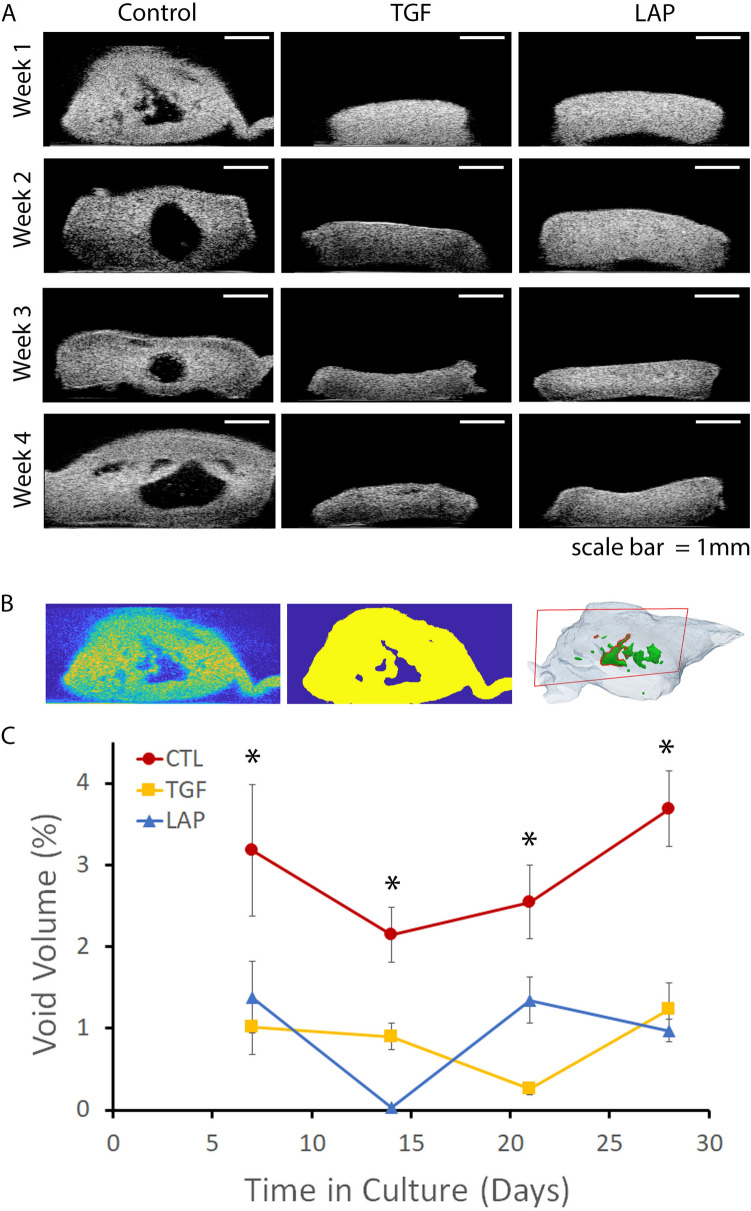
Quantitative UBM detected a reduction in void formation with LAP and TGF treatment. (**A**) Time course of representative UBM B-scan images from each treatment group over time. (**B**) Representative unprocessed (left), thresholded volume and void (middle), and 3D-rendered (right) images. (**C**) TGF and LAP treatment resulted in a significant reduction in void volume as compared to CTL at all time points.

UBM detected significant differences in void volume as well as sample dimensions (Fig. S1) with treatment and culture duration. Representative B-mode UBM images from the center of samples for the CTL, TGF, and LAP groups at each of the 4-week intervals showed large voids within the tissue engineered cartilage in the CTL group while both TGF and LAP appear more uniform throughout the culture duration (Fig. 3a). Image processing and 3D reconstruction of the UBM data (Fig. 3b) were performed to determine the percentage of void volume versus the total volume of each sample (Fig. 3c). While the void volumes did not increase over time (*p* = 0.21), there was a 3.8-fold reduction in void volume at day 28 in the TGF and LAP groups as compared to CTL.

### Destructive testing methods confirm LAP increased maturation and homogeneity of constructs

**Figure 4:**
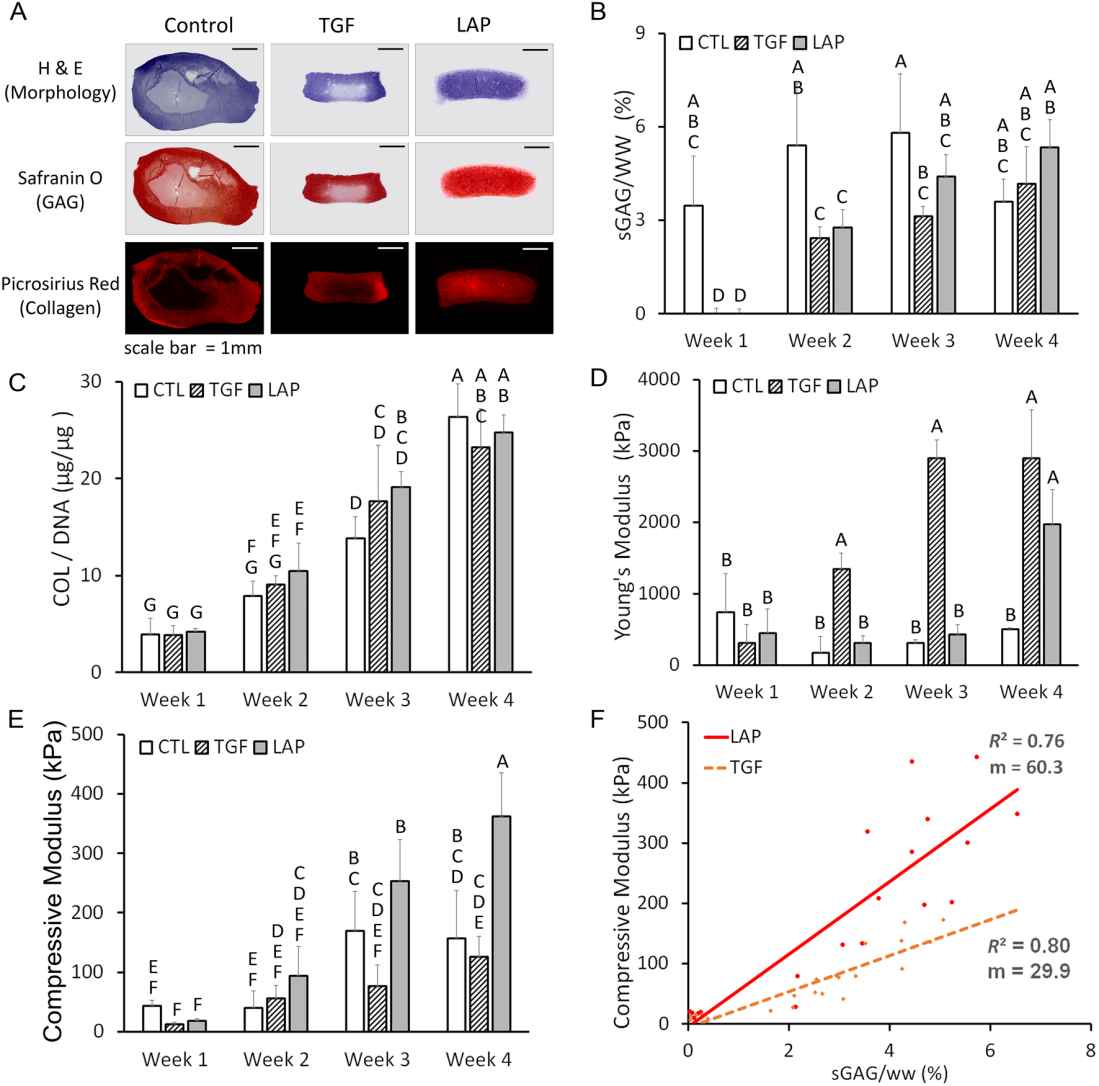
LAP increased maturation and homogeneity of constructs. (**A**) Histological staining of treatment groups at day 28 showed void formation and matrix inhomogeneity in CTL- and TGF-treated constructs, respectively. LAP treatment resulted in uniform staining. (**B**) GAG content decreased in both TGF and LAP treatments over CTL after cABC treatment at week 1 and recovered to CTL levels by Week 4. (**C**) Collagen content significantly increased over time in all conditions. (**D**) Tensile Young’s modulus significantly increased with TGF and LAP treatments over CTL at week 4. (**E**) Compressive modulus significantly increased with LAP treatment over CTL and TGF groups at week 4. (**F**) LAP-induced matrix homogeneity resulted in increased compressive modulus per GAG content as compared to TGF-treated constructs.

Neocartilage morphology was heavily dependent upon culture conditions. Histological staining at day 28 showed large void formation and matrix inhomogeneity in CTL- and TGF-treated constructs, while LAP treatment resulted in uniform safranin O and picrosirius red staining (Fig. 4a). At week 1, GAG content was decreased in both TGF and LAP treatments over CTL after chondroitinase-ABC (cABC) treatment, recovering to CTL levels by week 4 (Fig. 4b). Total collagen content significantly increased from week 1 to 4 in all conditions (6.7-fold; *p* < 0.001) with no significant differences between groups at any time point (Fig. 4c). Young’s modulus significantly increased from week 1 to 4 in TGF and LAP treatments (9.3- and 4.4-fold, respectively) (*p* < 0.0001) while CTL values remained constant resulting in a 479% increase in tensile stiffness with TGF and LAP treatments over CTL by week 4 (Fig. 4d). Compressive modulus values significantly increased from week 1 to 4 in all treatments (3.6-, 10.2-, and 20.7-fold, for CTL, TGF, and LAP, respectively) (*p* < 0.0001) resulting in a 189% increase in compressive stiffness in LAP treatments over TGF or CTL by week 4 (Fig. 4e). For constructs with equal amounts of GAG present, LAP-induced homogeneity resulted in increased compressive modulus as compared to TGF-treated constructs as shown by the higher slope (m values of the linear fit) of compressive moduli versus GAG content graphs (29.9 and 60.3 for TGF and LAP, respectively) (Fig. 4f).

### Optical assessment validated by strong correlations with destructive testing methods

**Figure 5:**
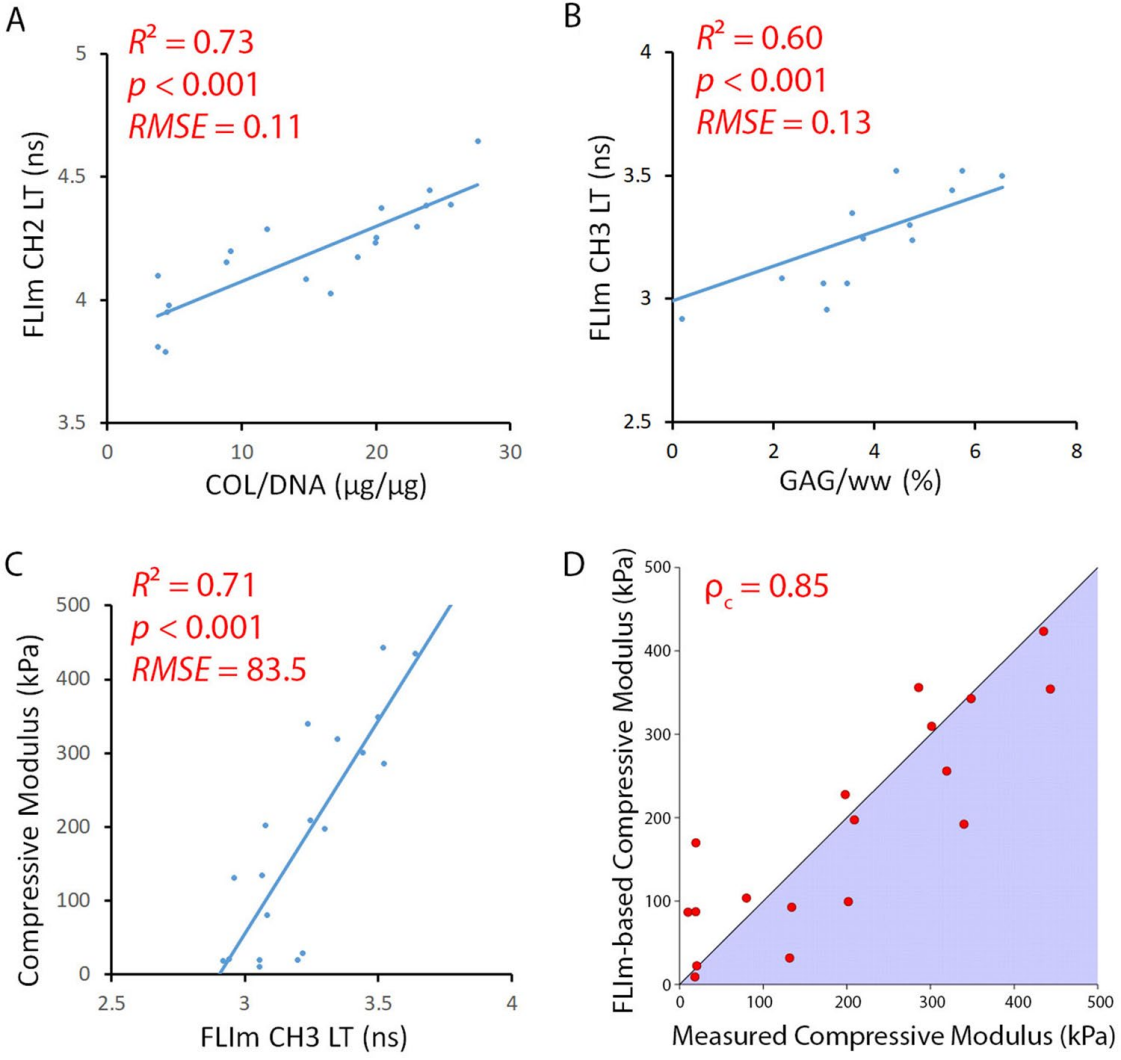
Optical assessment was validated by strong correlations with destructive testing methods. Destructive biochemical assays showed strong correlations between (**A**) collagen content and FLIm CH2 LT and (**B**) proteoglycan content and FLIm CH3 LT. (**C**) Destructive mechanical testing showed a strong correlation between FLIm CH3 LT and compressive modulus. (**D**) Comparison between calculated FLIm-based compressive moduli values and compressive moduli values measured via mechanical testing resulted in a high concordance correlation coefficient.

Nondestructive optical measurements showed strong correlations with biochemical assays and mechanical testing results. FLIm CH2 LT correlated with collagen content (*R*^2^ = 0.73; *p* < 0.0001) (Fig. 5a) and FLIm CH3 LT correlated with proteoglycan content (*R*^2^ = 0.60; *p* < 0.0001) (Fig. 5b). FLIm CH3 LT was found to have a strong correlation with compressive modulus (*R*^2^ = 0.71; *p* < 0.0001) (Fig. 5c). Comparison between calculated FLIm CH3 LT-based compressive moduli values and compressive moduli values measured during mechanical testing resulted in a high concordance correlation coefficient between the optical and gold standard testing methods (*p*_c_ = 0.85) (Fig. 5d).

### Homogeneity index for novel biochemical and structural characterization of constructs

**Figure 6:**
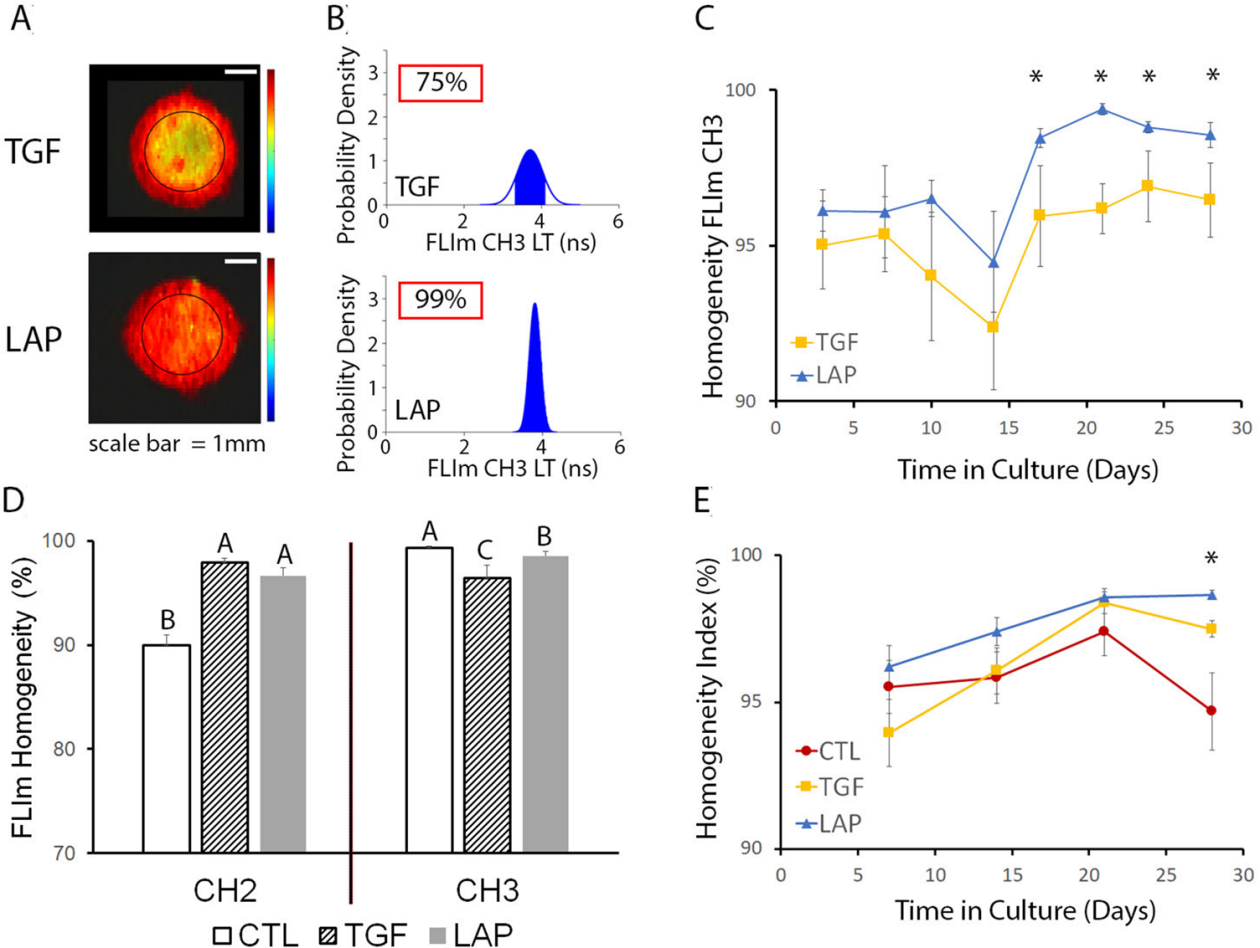
Imaging-based homogeneity index for the biochemical and structural characterization of constructs. (**A**) Representative *en face* FLIm LT images showing biochemical variations across the sample surface. (**B**) Example quantification of FLIm CH3 LT homogeneity from samples in panel A. (**C**) LAP treatment improved biochemical homogeneity from days 17–28 as compared to TGF. (**D**) FLIm LT homogeneity at day 28 varied with treatment in both CH2 and CH3. (**E**) An imaging-based homogeneity index combining structural and biochemical information showed significant improvements with LAP treatment at day 28 as compared to CTL and TGF.

FLIm LT values varied across samples to varying degrees, as seen in representative FLIm images of low and high biochemical homogeneity (TGF and LAP, respectively) (Fig. 6a). Evaluation of the distribution of LT values across the sample was calculated using statistical homogeneity theory to produce a quantitative percent homogeneity for each FLIm channel (Fig. 6b). For biochemical homogeneity in FLIm CH 3 LT (HF3), LAP had significantly increased HF3 over TGF from days 17–28 (*p* = 0.034) (Fig. 6c). Biochemical homogeneity in FLIm CH 2 LT (HF2) was calculated to fully characterize biochemical variation across the tissue and showed significant improvements in HF2 with both TGF- and LAP-treatments as compared to CTL (*p* = 0.0001) (Fig. 6d). An imaging-based homogeneity index (HI) that gives equal weight to the structural homogeneity data from UBM and the combined biochemical homogeneity data from FLIm CH2 and CH3 LT data showed that LAP had significantly higher total homogeneity than either TGF or CTL groups at day 28 (*p* = 0.008) (Fig. 6e).

## Discussion

The results of the present study demonstrate the applicability of a nondestructive imaging system, combining FLIm and UBM, to yield structure-function characteristics of tissue engineered cartilage over time. This multimodal system is potentially poised to provide full characterization of a neotissue product before releasing it for clinical use, thus, reducing biomanufacturing expenses significantly. The hypothesis that the multimodal system could quantify growth factor-induced differences in tissue maturation and homogeneity was supported by strong linear correlations between nondestructive and destructive tests. The hypothesis that an imaging-based homogeneity index combining biochemical and structural data could provide a novel assessment of tissue quality was supported by the ability of the homogeneity index to detect significant differences between treatment groups. The novel findings of this experiment include (1) sterile, repeated analysis of tissue engineered construct maturation and homogeneity over time, (2) image-based quantification of growth factor-induced increases in self-assembled cartilage homogeneity and mechanical properties, (3) validation of optical parameters with destructive assays, and (4) implementation of a novel imaging-based homogeneity index. The results of this study are particularly exciting because they serve as an initial step toward the development of a non-destructive continuous monitoring system for functional tissue engineering applications.

Nondestructive FLIm assessment, performed under sterile conditions, was capable of detecting and quantifying the increased matrix deposition and mechanical properties that accompany self-assembled cartilage formation^12^. The strong correlations between FLIm CH3 LT and GAG content and between FLIm CH2 LT and collagen content make a strong case for nondestructive quantification of biochemical content in cartilage. Structure-function relationships of articular cartilage^13^ allow mechanical properties to be inferred from the FLIm-derived biochemical composition, resulting in (1) strong correlations between optical assessment and compressive properties and (2) strong concordance between compressive moduli calculated from FLIm CH3 LT measurements and those obtained via a mechanical tester. Repeated exposure of individual samples to UV light (355 nm) did not significantly alter any of the measured biochemical, optical, or mechanical properties, suggesting that samples could be evaluated repeatedly in a research setting or as a quality control step prior to implantation. The relative uniformity in the biochemical composition in the tissue engineered constructs reduced any bias created by comparing FLIm surface measurements ($\sim$300 µm depth) to full thickness biochemical and mechanical properties. In contrast, native cartilage has significant depth-dependent variation in biochemical composition which cannot be captured by surface measurements. When we previously subjected native cartilage tissue to enzymatic treatments to deplete matrix content, we found lower than expected differences in fluorescence signal in cartilage imaged perpendicular to the tissue surface as compared to cartilage imaged in cross-section^5^. Depth-dependent properties and the presence of additional sources of tissue auto-fluorescence, including the extra-cellular matrix protein elastin^14^, will need to be considered before using this technology in other tissue engineering applications.

Large LAP-induced increases in compressive modulus over CTL or TGF groups may be partially attributed to a more uniform distribution of proteoglycan throughout the tissue and a reduction in void volume which decrease mechanical properties^15^. While collagen content did not differ among groups at each week, the increased tensile modulus in the LAP and TGF groups was likely due to increased collagen crosslinking density, resulting from exogenous lysyl oxidase like-2 (LOXL2), as shown previously^16^. At week 4, LAP constructs possessed Young’s modulus (3000 kPa), compressive modulus (350 kPa), collagen content (14%/ww), GAG content (6%/ww), and cell density values (10k cells/$\hbox {mm}^3$) on the order of native tissue values^17–19^.

The reduction in void formation with LAP and TGF groups compared to CTL may be attributed to the combination of collagen matrix compaction from cABC-induced GAG depletion and enhanced collagen crosslinking from LOXL2^20^. The resulting collagen network has increased fiber diameter, density, and tensile properties^20^, all of which resist swelling forces and void formation. The ability of UBM to nondestructively evaluate construct voids demonstrates the potential to remove defective samples during production to save valuable resources.

The development of an imaging-based homogeneity index, combining UBM data acquired throughout the depth of the sample and FLIm parameters from the surface layer of the sample, provides a single value to compare total sample homogeneity among groups. The homogeneity index provides a novel method of assessing tissue quality, currently missing in destructive testing methods. Histological analysis allows the visualization of voids or matrix homogeneities at discrete points, but it precludes evaluation of the entire clinical sample prior to implantation due to its destructive nature. In addition, homogeneity plays a role in the accurate assessment of mechanical properties because the continuum mechanics models used in characterizing tissue assume a homogeneous matrix. Tissue inhomogeneities violate this assumption resulting in erroneous biomechanical assessment.

In summary, we developed a nondestructive, multimodal imaging system to monitor the growth of tissue engineered articular cartilage in a longitudinal manner. The multimodal system measured the growth factor-induced improvements in tissue homogeneity and mechanical properties of self-assembled cartilage that resulted in native tissue properties. Our findings suggest that the multimodal system is effective in providing quantitative monitoring of the growth, quality, and production of neotissues. The methods presented here were demonstrated using cartilage as a model system. The multimodal system can also be adapted to other tissue engineering and regenerative medicine applications.

## Materials and methods

### Chondrocyte isolation

Articular cartilage was harvested from the femoral condyles of five juvenile bovine stifle joints (Research 87, Boston, MA), minced, and digested in Dulbecco’s Modified Eagle Medium (DMEM) with high glucose/GlutaMAX$^\mathrm{TM}$-I (Life Technologies, Grand Island, NY) with 0.3% collagenase type II (Worthington, Lakewood, NJ), 5% fetal bovine serum (FBS) (HyClone, GE Healthcare Life Sciences, Marlborough, MA), and 1% penicillin/streptomycin/fungizone (P/S/F) (Lonza, Basel, Switzerland) for 18h on an orbital shaker at 37 $^\circ$C. Following digestion, the cells were collected, pooled, filtered through 70 µm cell strainers, and washed three times with DMEM.

### Self-assembled construct culture

Constructs were formed using the self-assembling process, as described previously^21^. Briefly, $4 \times 10^6$ chondrocytes were suspended in 100 µL of control medium (CTL) consisting of DMEM with 1% ITS+ premix (BD Biosciences, Bedford, MA), 1% non-essential amino acids (NEAA) (Life Technologies), 50 µg/mL ascorbate-2-phosphate (Sigma-Aldrich, St. Louis, MO), 40 µg/mL L-proline (Sigma-Aldrich), 100 µg/mL sodium pyruvate (Sigma-Aldrich), and 100 nM dexamethasone (Sigma-Aldrich), and 1% P/S/F. Cell suspensions were seeded in 5 mm diameter, 2% agarose wells in 24-well plates (Costar, Corning, NY). After 4 h, 400 µL of control medium were added to each well. Self-assembled constructs were cultured for 4 weeks in (1) CTL medium only (CTL) or TGF, cABC, and LOXL2 (TCL)-treated^22,23^ with either (2) active TGF-$\beta$1 (TGF)(Propotech, Rocky Hill, NJ), or (3) recombinant human LAP TGF-$\beta$1 (LAP) (R &D Systems, Minneapolis, MN)^11^ applied at 10 ng/ml for the entire culture duration. In addition to TGF supplementation, TCL-treatment also consisted of cABC applied at 2 U/ml for 4 h at day 7^24^, and 0.146 mg/mL hydroxylysine (Sigma-Aldrich), 0.0016 mg/mL copper sulfate (Sigma-Aldrich), and 0.15 µg/mL of LOXL2 (SignalChem, Richmond, British Columbia, Canada) added for weeks 2–3^25^. All constructs were removed from agarose wells at day 5 and 1 mL of fresh media was exchanged daily.

### Nondestructive assessment of self-assembled articular cartilage

A multimodal tissue diagnostic system that combined the two complementary techniques of FLIm and UBM^26^ (Fig. 1b) was used to make a nondestructive assessment of the cartilage constructs twice per week for 28 days (n = 6/condition) under sterile conditions. In addition, matched samples were assessed nondestructively (FLIm-UBM), and destructively for their biochemical content (collagen and proteoglycan), mechanical properties (tensile and compressive), and by histological staining weekly. The FLIm system and analysis has been previously described^16^. Briefly, a pulsed 355 nm microchip laser (STV-02E-1x0, TEEM photonics, Grenoble, France) was used to generate sample autofluorescence through a flexible fiber-optic cable (10 m length $\times$ 400 µm diameter)(Molex, Lisle, IL). To allow sterile monitoring of a single sample over time, a three-axis digital translation stage (LP28, Parker, Cleveland, OH) was housed within a biosafety cabinet (Fig. 1c) and used to scan the fiber across the surface of the sample. The sample top/bottom (media/substrate) orientation was preserved throughout the experiment to keep measurements as consistent as possible. The distal tip of the fiber was positioned  1 mm above and perpendicular to the sample surface. Each sample was placed in a sterile 35 mm glass bottom dish (MatTech Corporation, Ashland, MA) in phosphate buffered saline at room temperature. Fluorescence emission was separated into four spectral bands (CH1 = 375–410 nm, CH2 = 450–485 nm, CH3 = 532–565 nm, CH4 = 595–660 nm) using a custom-built wavelength selection module, multiplexed onto a single photomultiplier tube (PMT) (R5916U-50, Hamamatsu, Bridgewater, NJ), and digitized using a high-speed data acquisition board (DAQ) (PXIe-5185, National Instruments, Austin, TX) with a temporal resolution of 80 ps. This enabled complete mapping of the surface of the sample in under 1 minute at a resolution of 20 µm/pixel. The mean and standard deviation of the intensity-weighted average LT in each spectral band were calculated from circular regions of interest. The attenuation of 355 nm light in tissue limited the penetration depth of FLIm to 250–300 µm.

### Ultrasound imaging and volumetric measurements

A 42 MHz ± 40% ultrasound transducer (4 mm aperture; 6 mm focal depth) (NIH UTRC, USC, CA) was raster scanned using the 3-D motorized translation stage. Pulsing/receiving was performed with a monocycle generator (Avitech AVB2-TE-C), 30 dB amplifier, and analog band-pass filter (10–100 MHz). Pulse-echo were sampled at 400 MHz using a PCI digitizer (Gage CS12400). A total of 32 averages were acquired at each scan location and spacing between frames was 200 µm. For B-mode image reconstruction, signals were band-pass filtered from 20–60 MHz, envelope detected, and log-compressed. Two-dimensional interpolation was used to yield 5 $\times$ 5 µm pixel resolution and 8-bit images were generated with 60 dB dynamic range. Void volume and total sample volume estimations were calculated from B-mode images to determine percent void volume. Briefly, For total volume segmentation, images were speckle reduced^27^, binarized using Otsu’s method^28^, and voids were flood-filled. For void segmentation, images were contrast-enhanced and binarized using an adaptive threshold. Binary mask interpolation was performed for both total sample and void masks across frames using a factor of 20 to render volumetric images. Total sample and total void volume were determined by voxel counting, and void volume (%) was calculated.

### Homogeneity index

Homogeneity from the structural UBM measurements (HS) was defined as 100 - void volume (%). Statistical homogeneity theory^29^ was used to determine FLIm LT homogeneity for spectral bands CH2 and CH3 (HF2 and HF3, respectively) based on the area under the probability density function (PDF) in the range of ± 0.1 x average LT value and expressed as a percentage (area under the entire PDF equals 100 %). The homogeneity index (HI) was defined as a 1:1 combination of the biological and structural homogeneity values as follows:

$$\begin{aligned} HI = \frac{1}{2} \left( HS + \frac{1}{2} \left[ HF2 + HF3\right] \right) \end{aligned}$$

A perfectly homogeneous material would score an HI of 100%.

### Biochemical analysis and histology

For biochemical analysis, tissue samples were measured to obtain wet (ww) and dry (dw) weights. Lyophilized samples were digested in papain for 18 h at 60 $^\circ$C, as previously described^30^. Sulfated GAG content was assayed using the Blyscan Glycosaminoglycan Assay kit (Biocolor, Westbury, NY) and total collagen content was quantified using a chloramine-T hydroxyproline assay (Biocolor)^31^. For histological evaluation, samples were fixed in 10% neutral buffered formalin, paraffin embedded, sectioned at 10 µm and stained with hematoxylin and eosin (H &E) for general morphology, safranin O for glycosaminoglycans, or picrosirius red for total collagen following routine procedures. Picrosirius red stain was visualized under fluorescence light microscopy using a standard filter set (Rhodamine: Ex: 538–562 nm; Em: 570–640 nm)^32^.

### Compressive and tensile mechanical testing

Compressive properties were quantified as previously described^33^. Briefly, a 2 mm cylindrical punch from each construct underwent stress relaxation testing in unconfined compression. Force-displacement data was recorded, converted to stress-strain based on sample dimensions, and all data were analyzed using KLM biphasic theory^34^ in Matlab (Mathworks, Natick, MA). Tensile testing was conducted using a uniaxial materials testing machine (Test Resources, Shakopee, MN) as previously described^20^. Briefly, cartilage samples were cut into dog-bone shaped tensile specimen and the sample thickness and width were measured via ImageJ software (NIH, Bethesda, MD)^35^. A uniaxial strain to failure test was conducted at a strain rate of 1% of the gauge length per second. Young’s Modulus was calculated by least squares fitting the linear portion of the resulting stress-strain curve in Matlab (Mathworks).

### Statistical analysis and validation

All evaluations in this study were performed using n = 6 matched samples per treatment group on JMP v13 (SAS Institute, Cary, NC). Data normality was tested using the Shapiro-Wilk goodness of fit test. Statistical analysis of groups was performed using one-way ANOVA with Tukey’s post-hoc analysis. Bar chart data are presented as the mean standard deviation with significant differences (p < 0.05) indicated by bars not sharing the same letter. Significant differences in treatment groups at each time point are presented by an asterisk (*) in longitudinal data. Correlations between bivariate parameters of each treatment were modeled separately to determine which outcome parameter correlated with which biochemical component using simple linear least squares regression analysis. Multivariable modeling was performed to determine the significance of repeated FLIm analysis on outcome variables. Lin’s concordance correlation coefficient was calculated using NCSSv12 statistical software (NCSS, LLC, Kaysville, Utah).

## Supplementary Information

Supplementary Information 1.

## Data Availability

All data generated or analyzed during this study are included in this published article.
