# Supplementary material for: Non-destructive, continuous monitoring of biochemical, mechanical, and structural maturation in engineered tissue
Source: Sci Rep. 2022 Sep 28;12:16227. doi: 10.1038/s41598-022-18702-x (PMC9519952; doi:10.1038/s41598-022-18702-x)
Supplement: Supplementary file 1 — Supplementary Information 1. [file 41598_2022_18702_MOESM1_ESM.pdf]

Supplementary Material

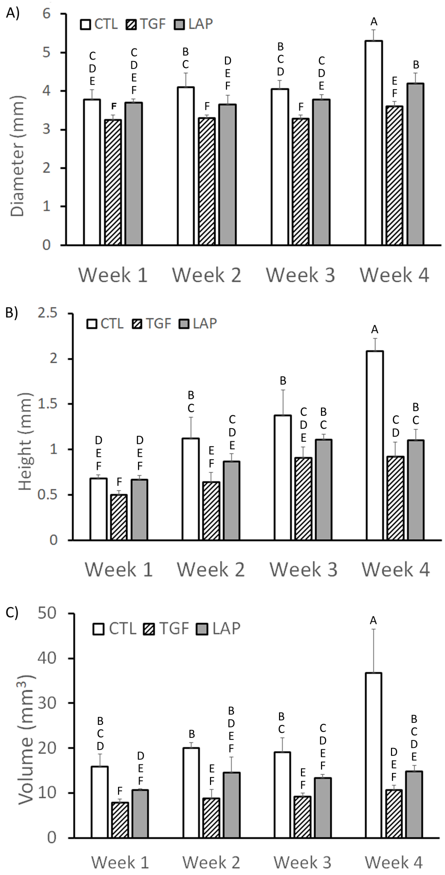

Supplementary Figure 1 : Effect of growth factors on the dimensions of self-assembled articular cartilage constructs over time. Average sample diameter (A), average height (B), and 3D-rendered volume (C) of self-assembled cartilage in CTL, TGF, and LAP culture conditions over four week time course. Statistical analysis of groups was performed using one-way ANOVA with Tukey's post-hoc analysis. Bar chart data are presented as the mean ± standard deviation with significant differences ($p<0.05$) indicated by bars not sharing the same letter.
